# Knowledge management and knowledge brokering in the Health Promotion Offices in Hungary: a qualitative study

**DOI:** 10.3389/fpubh.2025.1588370

**Published:** 2025-06-09

**Authors:** Attila Virág, Csaba László Dózsa, Borbála Cseh, Blanka Túri, Rita Teller, Gergő Túri

**Affiliations:** ^1^Institute for Entrepreneurship and Innovation, Corvinus University of Budapest, Budapest, Hungary; ^2^Department of Theoretical Health Sciences, University of Miskolc, Miskolc, Hungary; ^3^Synthesis Health Research Foundation, Budapest, Hungary; ^4^Faculty of Humanities, Eötvös Loránd University, Budapest, Hungary; ^5^Epidemiology and Surveillance Centre, Semmelweis University, Budapest, Hungary

**Keywords:** knowledge management, knowledge brokering, evidence-based decision-making, health promotion, program evaluation, stakeholder engagement

## Abstract

**Background:**

The Health Promotion Offices (HPOs) are essential institutions in the Hungarian public health system, providing public health services at the community level, operating intersectoral partnerships, and performing knowledge management and knowledge brokerage (KM/KB) functions. The study aimed to map the knowledge, experiences and practices of HPOs in the field of KM/KB and evidence-based decision making.

**Methods:**

The qualitative research study used semi-structured individual interviews with HPO members to collect their knowledge, experiences and insights regarding knowledge management, knowledge brokering and evidence-based decision-making. Twenty-two interviews were conducted in the summer of 2023, and a qualitative content analysis method was used to analyze the interview transcripts.

**Results:**

The activities of HPOs are multifaceted, encompassing various KM/KB elements. While HPOs are typically involved in knowledge acquisition, storage, communication and exchange, the evaluation of the social and economic benefits of public health programs and services is an area that requires further development. HPO members have differing views on their role in evidence-based decision-making, but many believe that HPOs actively support local decision-makers. According to HPO members, they are most active as knowledge brokers in community health planning. The barriers to KM/KB are decision-makers disinterest and different organizational or personal motivations. Factors that support KM/KB are the local embeddedness of HPOs, their advocacy practices and their approach to health in all policies.

**Conclusion:**

The HPOs are involved in KM/KB activities and support a more pronounced presence of these functions in their portfolio. However, this requires the improvement of the current funding methodology, the establishment of KM/KB protocols and training, and clarifying the roles and responsibilities of HPOs in the legislation.

## 1 Introduction

The role and benefits of knowledge management (KM) and knowledge brokering (KB) functions and activities in public health have been an important area of research for decades (1–7). Knowledge management is defined in many different ways in the international literature (8–11). Some authors define knowledge management as knowledge exchange processes in formal and informal networks within an organization. In contrast, others define it as the set of knowledge exchange methods in formal and informal networks within an organization or even across sectors. Knowledge management is the set of processes that enable the acquisition, storage, dissemination, use and development of knowledge, enabling one or more members of an organization to effectively apply knowledge and improve decision-making mechanisms as part of a network to solve complex problems (3, 11).

Several terms are used in the international literature as synonyms for knowledge brokers as individuals, such as innovation brokers, knowledge intermediaries, technology brokers, and change agents (5, 12–15). If knowledge brokers are defined as organizations, they are also referred to as intermediary firms, boundary organizations or bridging institutions. Knowledge brokers, whether individuals or organizations, act as intermediaries for interpreting, sharing and applying knowledge, information and experience between two or more actors (such as individuals, organizations, communities or networks).

Knowledge brokers can play an essential role in the knowledge-producing research community and the knowledge-using decision-makers, stimulating the emergence of new information, collaborative knowledge exchange, and evidence-based approaches (16–19). Knowledge Brokers’ activities include building and managing partnerships and networks; identifying and connecting key actors; identifying, evaluating and analyzing relevant information; supporting communication and information sharing; and supporting evidence-based decision-making (12, 14, 18, 20).

Hungary, a Central European country of 9.5 million people, is a member of the European Union and faces challenges similar to those developed countries face regarding their health systems and the health status of their populations (21). According to several studies, life expectancy at birth in Hungary is below the EU average and morbidity and mortality rates due to several chronic diseases are higher than the EU average (22–25). In response to the public health challenges, 110 HPOs have been established in Hungary in several phases since 2008, providing free individual-oriented health promotion and preventive services, and community health services in almost two-thirds of the Hungarian districts (26). The HPOs are building a network of partnerships with experts and leaders from different sectoral organizations, for-profit and non-governmental organizations and municipalities. They also carry out health communication activities and participate in developing and implementing community health plans.

The HPOs have an average of 7.6 staff, and the network employs more than 800 professionals, a large proportion of whom have a health and public health background and a smaller proportion with a background in economics, social sciences and social work. The HPOs are owned by healthcare providers, universities or municipalities, and the ownership background leads to different practices in the financing, service portfolio and operating model of the HPOs. A detailed description of the services provided by HPOs and how HPOs are managed and funded has been described in a previous research paper (26). HPOs have become an essential part of the Hungarian public health system over the last 10 years, with several KM/KB elements identified in their professional portfolio.

The specific objectives of our research were:

1. To map the knowledge and practices of HPOs in the field of KM/KB;
2. To identify factors that support or hinder HPOs in implementing KM/KB activities;
3. To identify the experiences of HPOs as knowledge brokers in implementing evidence-based decision-making at the local level.

## 2 Methods

### 2.1 Research design

The qualitative research study used semi-structured individual interviews with HPO members to collect their knowledge, experiences and insights regarding knowledge management and knowledge brokering (27). The study was designed to meet the COREQ criteria for reporting qualitative research, provided in Appendix 1.

### 2.2 Participants and recruitment

The research team selected specific individuals for interviews based on purposeful criteria. The aim was to interview at least 20 persons and ensure sufficient variation via the following criteria: (a) at least two HPO members from every Hungarian region, (b) at least three HPO members from every pre-defined age-groups, (c) at least six HPO members from rural area, six from urban area and six from mixed area (urban and rural), (d) at least HPO professional leaders, five economic managers, five public health experts, and five healthcare experts. The health expert HPO members had a medical or nursing degree, and the public health expert HPO members had a degree in health promotion, public health or epidemiology.

HPO members were eligible to participate in the interviews if they were members of a HPO for at least 2 years, were willing to provide insights from their experiences regarding knowledge management and knowledge brokerage, and provided informed e-consent. Possible participants were identified by the research team members using the HPOs online websites. HPO members from different regions and practice types were invited via an electronic notice sent to their work email to take part in an individual interview. The willingness to participate could be indicated by filling in a participant information sheet about general information on the HPO, such as, geographical region, area, and suitable time for the interview. Participants were contacted via email and informed e-consents were collected by return email before the interviews.

### 2.3 Data collection and processing

The research team has developed a semi-structured interview guide in accordance with the research objectives. Thematic fields were (a) knowledge and familiarity of KM/KB concepts; (b) current KM/KB activities and experiences in HPOs; (c) opportunities for broad implementation of KM/KB functions, evidence-based decision making; (d) barriers and facilitators to KM/KB activities. Questions were asked by the Interviewer but not provided to participants. During the development of the interview guide, it was pilot-tested with three Health Promotion Office members. The test interviews conducted by the research team during the pilot test were not included in the analysis.

All interviews were conducted from June to August 2023 by GT, BC, RT and CLD. All researchers had prior experience conducting interviews in qualitative studies that evaluated the implementation of healthcare interventions. All interviews were recorded using Zoom teleconference. Interviews were transcribed verbatim from the audio by GT, BC, RT, and CLF using Microsoft Word, and GT checked transcripts for consistency. The semi-structured interview guide is provided in Appendix 2.

In qualitative research, data saturation indicates the point at which collecting more data is unlikely to reveal new insights. The literature suggests that this can generally be achieved through 7 to 18 interviews (28, 29). To assess data saturation in our study, we followed the methodology of Guest and colleagues, using a threshold of 5% for new information (28). We ultimately conducted 22 interviews, which were sufficient to achieve thematic saturation, ensuring that a comprehensive range of relevant themes was captured in our findings.

The study used Carter et al.’s methodology for data triangulation, revealing consistent interview findings (30). Four experts from various fields conducted the interviews, while two experts coded the transcripts and developed the coding system. Interviews were conducted with HPO members of different age groups, genders, professional roles, and geographical regions. The entire research team participated in exploring the potential interpretations of the data. The methodological triangulation encompassed comprehensive interviews, detailed field notes, and a thorough collection of pertinent legislation, policy documents, and literature on the subject, ensuring a robust analysis of the topic.

### 2.4 Analysis

The analysis used the Directed Content Analysis method as described by Hsieh & Shannon (31). This method takes a deductive approach and uses an existing theory or framework to develop the initial codes. The WHO Health Systems Framework was chosen as the first step of the Directed Content Analysis because it allowed for a broad, systemic analysis of the research topic and categorizing the results into major building blocks or subsystems (32). This framework supported identifying challenges and opportunities for improvement by the subsystem to facilitate more effective KM/KB activities and evidence-based decision-making by HPOs. In the second step, the initial codes (a priory coding system) were formulated based on the six main building blocks of the WHO framework as follows: financing, physical resources, human resources, governance and leadership, information and research, and service delivery. In the next step, the interview transcripts were coded, first with the predefined coding system and then with new categories based on new themes emerging in the transcripts. Each transcript was coded at least twice by GT, BC, using Atlas.ti 22 software, and CLD validated the results. In the fourth step, the research group analyzed and interpreted the relationship and meanings between codes and categories. In order to foster a collective understanding, in-depth discussions focused on consensus-building were organized. These discussions aimed to thoroughly evaluate and agree upon the overarching themes of the research. Throughout this process, the entire research team collaborated to interpret the results.

### 2.5 Ethical considerations

Aggregated sociodemographic information was collected from interviewees. The participants provided written informed consent to participate in this study, signifying their willingness to participate and their permission for the interviews to be recorded. Prior to conducting the interviews, participants received both oral explanations and written documentation outlining the study’s purposes, procedures, potential risks, benefits, their voluntary involvement, and the confidentiality of their input. To protect participant’s identities and maintain anonymity, their names were replaced with unique identifiers (ID1-ID22). Participants were made aware that they could withdraw at any time. All audio recordings and transcripts were stored on a password-protected, encrypted server accessible only to the research team. This research project was deemed exempt in accordance with local legislation by the Research Ethics Committee of Synthesis Health Research Foundation because (1) the participants were interviewed in their profession; (2) patients or clinical data from patient documentation were not included in the study. All original data, including audio files and de-identified transcripts will be permanently destroyed 2 years after the completion of data analysis using certified digital file shredding software and secure document disposal methods.

## 3 Results

In total, 33 HPO members were invited, and 22 HPO members participated in the study (Table 1). 11 HPO members did not respond to the invitation. The audio interviews were an average of 55 min. Participants were from a diverse age groups, half were female (11/22, 50%) and the four profession type were represented nearly equally. The largest proportion of interviewees were from a HPO of an urban area. At least two participants were recruited from each region of Hungary. Most interviewees (83%, 18 out of 22) had encountered the terms “knowledge management” and “knowledge broker” in their professional careers and possessed varying degrees of understanding regarding these terms.

**Table 1:** Characteristics of interviewed participants (*n* = 22).

Age groups (years)	20–29	3
	30–39	6
	40–49	5
	50–59	5
	60–69	3
Gender	Female	11
	Male	11
Professional role	Professional leader	6
	Economic manager	5
	Public health expert	6
	Healthcare professional	5
Region	Western Transdanubia	3
	Southern Transdanubia	2
	Central Transdanubia	2
	Central Hungary	3
	Budapest	4
	Southern Great Plain	3
	Northern Great Plain	3
	Northern Hungary	2
Area	Urban	10
	Rural	6
	Mixed	6

Sources: Authors.

The main themes identified in the analysis were grouped according to the blocks of the WHO health systems framework (Table 2). The following section describes these themes in more detail.

**Table 2:** Main themes grouped according to the blocks of the WHO health system framework.

Building block	Themes
Financing	Funds dedicated to KM/KB in the HPOs budget
	Adaptation and transfer of innovative financing methods
Physical resources	Physical resources improvements to support KM/KB
Human resources	HR developments supporting the KM/KB functions
Governance and leadership	A variety of operational and governance models for HPOs
Information and research	Evaluation of services and programs
	Evidence-based decision-making
Service delivery	KM and KB as existing practices in HPOs
	Advocacy and health planning
	Interests and motivations of decision-makers
	Local embeddedness and a health in all policies approach

Sources: Authors.

### 3.1 Financing

#### 3.1.1 Funds dedicated to KM/KB in the HPOs budget

Most interviewees expressed that the HPOs’ tasks currently include networking and advocacy, but their primary focus is providing health promotion services. The HPOs’ current funding system is suitable for funding health promotion services and programmes, but knowledge brokering tasks could be monitored and accounted for using a different logic. A more prominent role for KM/KB functions may require dedicated resources in the HPOs’ budgets.

"I believe it would be beneficial for the profile of an HPO organization or the roles of HPO employees to include more tasks related to knowledge brokering. However, implementing these tasks would require a different funding method and a separate budget to support them." ID 18

#### 3.1.2 Adaptation and transfer of innovative financing methods

As in developed countries, financing public health services and programs is a central issue of the scientific dialogue in Hungary. Some of the interviewees highlighted that the adaptation and dissemination by HPOs of innovative financing techniques that would attract resources from new actors or areas into public health could facilitate the financing and implementation of health promotion programs. Some interviewees see knowledge brokering as an opportunity to disseminate innovative financing techniques, but also a need for appropriate legal regulation and methodological underpinning in this area.

*“A few years ago, we were involved in a project to learn about financing methods such as social impact bonds and outcome-based financing. HPOs could play a key role in disseminating these methods, even as knowledge brokers” ID 1.*

### 3.2 Physical resources

#### 3.2.1 Physical resources improvements to support KM/KB

The majority of interviewees also believe that infrastructure and tools need to be developed to present themselves as credible and accepted knowledge brokers to the leaders of other organizations. To fulfil their knowledge management tasks, HPOs need more modern office infrastructure and tools, which would support the receptiveness and willingness of local policymakers and managers of companies and institutions to cooperate with HPO staff.

*“I think that to be a knowledge broker, you need representative spaces and smart tools such as a well-equipped meeting room, digital projector, modern laptop and car, and presentation tools that not all HPOs currently have. A mayor or a company director is more likely to sit down with me if they see that the organization I represent has the resources to do the job” ID 3.*

### 3.3 Human resources

#### 3.3.1 HR developments supporting the KM/KB functions

The skills and professional backgrounds of HPO staff are very diverse. While some HPOs have staff who can effectively perform KM/KB tasks and functions, other HPOs require HR development to perform these functions. Among the interviewees, recruiting new staff and retraining some existing staff was also raised as an option to enable the HPO to fulfil its role as a knowledge broker.

*“I believe that to implement the tasks associated with knowledge brokering successfully; you need broad and intersectoral knowledge and years or decades of experience. Not all HPOs have such a background, so you must train such professionals or find resources to recruit new staff” ID 7.*

### 3.4 Governance and leadership

#### 3.4.1 A variety of operational and governance models for HPOs

The current operation and management of HPOs are heterogeneous, as HPOs are owned by different types of organizations with different motivations, such as hospitals, municipalities or universities. Ownership can affect the service portfolio of HPOs (medical or public health focus) and the way services are delivered (services targeted at individuals or community programs are more emphasized). Ownership types also lead to differences in organizational and funding methods. HPOs also shape their service portfolios according to the needs of local communities, which also results in heterogeneous practices. Thus, some HPOs emphasize KM/KB functions and elements in their day-to-day operations, while others focus less on KM/KB functions and elements.

*“I work in a hospital-owned HPO, where we provide services to the population, mostly targeting individuals. I can't think of the HPO or myself as a knowledge broker, and it would be a bit of unfamiliar field for me” ID 9.*

*“As the head of a municipally owned HPO, I see that we are currently actively engaged in KM and KB activities, which is facilitated by the fact that there is an active partnership between the municipality's management and other municipally owned health and social care providers. In the planning and implementation of local public health programs, the HPO thus plays an active role as a facilitator and as an actor that shares knowledge and information, interprets it and supports its use” ID 12.*

### 3.5 Information and research

#### 3.5.1 Evaluation of services and programs

According to the majority of the interviewees, the evaluation of programs and services is currently missing from the general tasks of HPOs among the KB functions, and it would be worth adapting this to the functioning of HPOs. Evaluating the social and economic impact of services and programs provided by HPOs and sharing the results of analyses is considered important because it can inform the development of services and support the dissemination of good practices within the HPO network and among organizations within and outside the public health sector.

*“HPOs do not currently have a methodology to assess the health or economic impact of the programs they or other providers provide. At professional conferences, we sometimes see case studies of programs delivered by HP. However, I don't think anyone is evaluating them with a widely accepted, standard set of criteria, which would provide important national or local information to know how useful what we or other colleagues are doing” ID 14.*

#### 3.5.2 Evidence-based decision-making

The interviewees had different views on the role of HPOs in supporting evidence-based decision-making. According to half of the interviewees, HPOs, as a kind of knowledge brokers, currently support local or county decision-makers in implementing evidence-based programs and services that respond to community needs in the communities in their areas of operation. These interviewees highlighted that several projects have been implemented in recent years that have produced detailed descriptions in Hungarian of national and international programs with proven impact and guidance for their implementation. At the same time, other interviewees argued that evidence-based decision-making is less common at the level of smaller municipalities or even at the national level, which they identified as being driven by cultural and social reasons.

*“I currently see the HPO as a knowledge broker, linking leaders and experts from organizations in different sectors with researchers who create knowledge, and in doing so, we support the use of available resources to deliver programs at the community level that have a good chance of being of social benefit” ID 16.*

*“I think that while it would be desirable if decision-makers made evidence-based decisions about where to spend taxpayers' money, the reality is that this is often not the case. To change this, HPOs are not enough; they would also require a change in social and cultural norms, expectations of decision-makers, and the transparency of the decision-making process” ID 11.*

### 3.6 Service delivery

#### 3.6.1 KM and KB as existing practices in HPOs

HPOs carry out a variety of KM and KB activities in their operations, but this is still a less prominent element of their tasks. Most interviewees identified the acquisition, storage, communication and exchange of knowledge and information with actors within and outside the sector as an activity typical of the day-to-day practice of HPOs. Building and operating networks and partnerships was also identified as a typical practice. However, a less common practice among HPOs is analyzing and evaluating knowledge and information.

*“I believe that HPOs, in addition to individual and community health promotion services, also carry out several activities that can be identified as knowledge management or knowledge brokering. We have developed and operated an active network and partnership with local public health actors and decision-makers over the past years and have shared relevant knowledge and information with them on some platforms, supporting the development of their services and intersectoral programs” ID 18.*

#### 3.6.2 Advocacy and community health planning

Most interviewees identified the benefits of adapting KM and KB functions by HPOs in health planning and advocacy with decision-makers. Some HPOs have been actively involved in developing community health plans and implementing some programs in previous years. The processes of health planning, such as assessing and evaluating population needs, designing, implementing and evaluating evidence-based programs, and actively engaging communities and decision-makers, are tasks that fit within the knowledge brokering functions and can be carried out by HPOs. However, this may require additional training and resources, as well as clarification and updating of the legislation on the competencies of health planning actors. As they could help represent the interests and needs of different social groups and interpret scientific evidence to decision-makers, interviewees also considered knowledge brokerage functions helpful.

*“In my opinion, community health planning is the task for HPOs where they function best as a knowledge broker. In our municipality, the community health plan was completed two years ago. On several occasions, the HPO has been able to play an active knowledge-sharing role in the process, supporting decision-makers in identifying problems and designing evidence-based programs to respond to them” ID 10.*

#### 3.6.3 Interests and motivations of decision-makers

According to some interviewees, the lack of interest of decision-makers and the fact that decision-making processes may be influenced by different organizational or personal motivations and interests may be a barrier to knowledge brokering. The difficulty of achieving commitment to medium-and long-term local or national public health programmes from several politicians and stakeholders, as the social benefits can only be realized in a subsequent electoral cycle, was also mentioned as a barrier.

*“The work of a knowledge broker can be hampered by the fact that the decision-making processes of municipal leaders are often not transparent and can be influenced by different interests. Having a range of evidence-based programs and effective communication tools is useless if other actors or factors better influence the decision-maker” ID 4.*

*“In my view, a politician can only be persuaded by us to support a program that will have results before the next election” ID 17.*

#### 3.6.4 Local embeddedness and a health in all policies approach

According to the majority of interviewees, one of the factors supporting the activities of HPOs in KM and KB is their embeddedness in local communities and the public health focus of the organizations. HPO members have developed broad cross-sectoral partnerships over the past years and have active relationships with several local service providers, non-profit organizations, experts and decision-makers. Some of the interviewees also highlighted that the fact that HPOs have many years of practical experience in representing all health policy areas is a factor supporting the KB function. Some interviewees emphasized the need to enshrine in legislation the role and responsibilities of HPOs in developing and implementing local policies and action plans for health.

*“HPOs can potentially be effective knowledge brokers at organizational or individual level. They have an extensive network of partners in local communities and are active in knowledge sharing. With the proper training, I believe we could play a greater role in promoting health in all sectors at the local level” ID 20.*

## 4 Discussion

The activities of HPOs are multifaceted, encompassing various KM/KB elements. Like many North American and Western European public health organizations, HPOs are typically involved in knowledge acquisition, storage, communication and exchange (8, 16). Similar to the diverse international practice of knowledge brokering, we have identified the implementation of knowledge brokering functions at the organizational and personal levels (by HPO members) in HPOs (5, 11, 12, 15, 18). The international practice of knowledge brokering activities often includes evaluating information, knowledge, programs and services, which is an even less pronounced element in the case of HPOs but is identified as an area for improvement (3, 8, 11, 16).

Similar to the results of several international studies, we found that the effective implementation of KM/KB activities can be hampered by the lack of interest of decision-makers and by different organizational and personal motivations (33–36). Dageanis et al. cite the quality of social networks, collective empowerment, and the availability of scientific outputs as influencing factors (2). Dobbins et al. mention supporting infrastructure, among others, as a factor supporting KB (3). The issue of physical resources was also raised by interviewees in our study, suggesting that without adequate infrastructure and facilities, knowledge brokers may be less likely to project an image of credible expertise to decision-makers. The factors identified as supporting the KM/KB functions of HPOs are their embeddedness in local communities, their advocacy experience and their approach to health in all policies, which are also identified as essential factors in the international literature (33–36).

Several international studies have shown that knowledge brokers can play an important role in supporting evidence-based decision-making at local and national levels (15, 17, 18, 37). In our study, interviewees had mixed views on the involvement and role of HPOs in this process, but many felt that HPOs are currently active in supporting local evidence-based decision-making, most notably in community health planning. At the same time, others believe that this is less the case for the activities of local and national decision-makers, which were cited as being driven by cultural and social reasons. Our findings are similar to international studies that suggest that the adaptation of evidence-based decision-making within organizations may be influenced by a range of social, cultural and economic factors, which also require different approaches by knowledge brokers. Barbarczy et al.’s study suggests that it is important not only to have KM strategies within an organization but also to have the extent to which stakeholders adapt and apply them in day-to-day practice (38).

Our research findings resonate with conclusions drawn from various studies conducted in middle and low-income countries, highlighting a shared understanding across diverse contexts, where the main barriers to knowledge management are inadequate communication, conflicting and counter motivations, and insufficient leadership (39–41). To implement successful knowledge management processes, it is essential not only to overcome these barriers but also to develop the HR and methodological capacities of the knowledge broker. Similar to our research findings, a challenge identified in middle and low-income countries is the development of linkages and collaboration between stakeholders and researchers who generate scientific evidence (42, 43). However, the early involvement of decision-makers in certain research processes and the interpretation of their potential social and economic benefits can facilitate the uptake of evidence-based decision-making in all countries.

Our research has several limitations. First, the characteristics of qualitative research mean that the results are not necessarily generalizable to the entire HPO network. However, the study strictly adhered to the methodological requirements for qualitative studies, and interviewees were selected considering a range of perspectives and factors. A further limitation of our research is that, as participation was voluntary, there may have been selection bias among HPO members who had knowledge and information about KM/KB issues or were particularly interested in the topic.

## 5 Conclusion

The activities of HPOs are multifaceted, encompassing various KM/KB elements. HPO members have differing views on their role in evidence-based decision-making, but many believe that HPOs actively support local decision-makers. HPOs are most active as knowledge brokers in community health planning. The barriers to KM/KB are decision-makers’ disinterest and different organizational or personal motivations. Factors that support KM/KB are the local embeddedness of HPOs, their advocacy practices and their approach to health in all policies. It would also be essential to clarify the roles and responsibilities of HPOs in legislation, which would provide them with an appropriate legal basis to represent health concerns in the design, implementation and evaluation of various sectoral policies and action plans.

## Data Availability

The datasets presented in this article are not readily available because of the nature of the qualitative study method, and to protect study participants privacy. Requests to access the datasets should be directed to turi.gergo@semmelweis.hu.
