# Supplementary material for: Knowledge management and knowledge brokering in the Health Promotion Offices in Hungary: a qualitative study
Source: Front Public Health. 2025 Jun 9;13:1588370. doi: 10.3389/fpubh.2025.1588370 (PMC12183307; doi:10.3389/fpubh.2025.1588370)
Supplement: Supplementary file 1 [file Table_1.docx]

Appendix 1. Consolidated criteria for reporting qualitative research (COREQ) Checklist.

| **Domain 1: Research team and reflexivity** |  |
| --- | --- |
| *Personal Characteristics* |  |
| 1. Interviewer/facilitator: Which author/s conducted the interview or focus group? | Interviews were conducted by GT, or RT, or BC, or CLD. |
| 2. Credentials: What were the researcher’s credentials? E.g. PhD, MD | GT holds a BA in Sociology and an MSc in Health Economics.  RT holds a BA in Sociology and an MSc in Economics.  BC holds a PhD in Health Sciences  CLD holds an MSc in Economics and a PhD in Health Economics. |
| 3. Occupation: What was their occupation at the time of the study? | GT is an Epidemiologist and Health Economist, RT is a Researcher, BC is a Researcher, and CLD is a Health Economist. |
| 4. Gender: Was the researcher male or female? | RT and CB both identify as female, GT and CLD both identify as male. |
| *Experience and training* |  |
| 5. What experience or training did the researcher have? | GT has organized, supervised and taken notes for  >12 focus groups, led 5 focus groups and conducted  >40 individual semi-structured interviews with research participants.  RT has conducted  >20 individual structured interviews with research participants.  CB has organized, supervised and taken notes for > >5 focus groups and led 2 focus groups and conducted  > 23 structured interviews with research participants.  CLD has organized, supervised and taken notes for > 32 focus groups and led 10 focus groups and conducted  > 65 structured interviews with research participants. |
| *Relationship with participants* |  |
| 6. Relationship established: Was a relationship established prior to study commencement? | GT, RT, CB and CLD had no prior relationships with any of the participants who took part. |
| 7. Participant knowledge of the interviewer: What did the participants know about the researcher? e.g. personal goals, reasons for doing the research | None of the participants knew the interviewer prior to the interviews. All participants read and signed the consent form, so they knew the reasons and goals for conducting the interview. |
| 8. Interviewer characteristics: What characteristics were reported about the interviewer/facilitator? e.g. Bias,  assumptions, reasons and interests in the research topic | No characteristics were reported. |
| **Domain 2: study design** |  |
| *Theoretical framework* |  |
| 9. Methodological orientation and Theory: What methodological orientation was stated to underpin the study? e.g. grounded theory,  discourse analysis, ethnography, phenomenology, content analysis | Qualitative content analysis. |
| *Participant selection* |  |
| 10. Sampling: How were participants selected?  e.g. purposive, convenience, consecutive, snowball | Purposive |
| 11. Method of approach: How were participants approached? e.g. face-to-face, telephone, mail, email | Participants were invited via email. |
| 12. Sample size: How many participants were in the study? | 33 participants were invited 22 interviews were conducted |
| 13. Non-participation How many people refused to participate or dropped out? Reasons? | 11 Health Promotion Office member did not respond to the invitation. |
| *Setting* |  |
| 14. Setting of data collection: Where was the data collected? e.g. home, clinic, workplace | Online using Zoom teleconference |
| 15. Presence of non-participants: Was anyone else present besides the participants and researchers? | No |
| 16. Description of sample What are the important characteristics of the sample? e.g. demographic data, date Data collection | Demographic data |
| 17. Interview guide: Were questions, prompts, guides provided by the authors? Was it pilot tested? | Questions were asked by the Interviewer but not provided to participants. Interview guide was pilot tested with three Health Promotion Office members. |
| 18. Repeat interviews: Were repeat interviews carried out? If yes, how many? | No. |
| 19. Audio/visual recording: Did the research use audio or visual recording to collect the data? | Audio recording was used to collect data via Zoom teleconference. |
| 20. Field notes: Were field notes made during and/or after the interview or focus group? | Yes, after the interviews. |
| 21. Duration: What was the duration of the interviews or focus group? | 48 minutes |
| 22. Data saturation: Was data saturation discussed? | Yes |
| 23. Transcripts returned: Were transcripts returned to participants for comment and/or correction? | No |
| **Domain 3: analysis and findings** |  |
| *Data analysis* |  |
| 24. Number of data coders: How many data coders coded the data? | Two study members (GT and RT) coded the data with discrepancies checked by third (CLD) study member. |
| 25. Description of the coding tree: Did authors provide a description of the coding tree? | Yes |
| 26. Derivation of themes: Were themes identified in advance or derived from the data? | Yes, themes derived from the data. |
| 27. Software: What software, if applicable, was used to manage the data? | All interviews were transcribed verbatim from the audio by GT, RT, BC and CLD in Microsoft Word, thematic analysis produced with Atlas.ti 22 sotftware. |
| 28. Participant checking: Did participants provide feedback on the findings? | No |
| *Reporting* |  |
| 29. Quotations presented: Were participant quotations presented to illustrate the themes  / findings? Was each quotation identified?  e.g. participant number | Yes |
| 30. Data and findings consistent: Was there consistency between the data presented and the findings? | Yes |
| 31. Clarity of major themes: Were major themes clearly presented in the findings? | Yes |
| 32. Clarity of minor themes: Is there a description of diverse cases or discussion of minor themes? | Yes |
