# Supplementary material for: Knowledge management and knowledge brokering in the Health Promotion Offices in Hungary: a qualitative study
Source: Front Public Health. 2025 Jun 9;13:1588370. doi: 10.3389/fpubh.2025.1588370 (PMC12183307; doi:10.3389/fpubh.2025.1588370)
Supplement: Supplementary file 2 [file Table_2.docx]

Appendix 2. Interview Guide.

The interview guide has been translated from Hungarian.

**Introduction of the researcher**

**Brief information about the study**

You have been invited to participate in an online interview for the study on knowledge management and knowledge brokerages in the Health Promotion Offices in Hungary. An independent researcher from Semmelweis University/Eötvös Loránd University/University of Miskolc will conduct the interview.

**Main purpose of the research**

We would like to get a comprehensive picture regarding the following themes:

-What knowledge do you have about knowledge management, knowledge brokering?

-What activities related to knowledge management or knowledge brokering do you carry out in Health Promotion Offices?

-What factors hinder or support Health Promotion Offices to participate widely and effectively in knowledge management processes or to act as knowledge brokers in the public health network?

-Can knowledge brokers promote evidence-based decision-making at the municipal and district levels in planning, implementing and evaluating health-related interventions?

**Technical information**

Participation in the interview is entirely voluntary. You are under no obligation to answer all questions or to participate in the interview. Your decision to not answer a question or not to participate will be fully respected and will not result in any form of discrimination.

The interview, which will take 45-60 minutes, will be conducted with utmost confidentiality. The entire interview will be audio-recorded, but no one will be identified.

**Interview questions**

**Mapping knowledge on knowledge management and knowledge brokering**

- Have you encountered the terms "knowledge management" and "knowledge brokering" in your professional career? If so, explain what you know about this topic in a few sentences.

In the following, I will give a concise definition of knowledge management and knowledge brokerage based on international literature so that we can understand the same concepts in the rest of the interview.

**The definition of knowledge management**

Knowledge management is defined in many different ways in the international literature. According to some authors, knowledge management is a process within an organization that aims to use knowledge more effectively and improve decision-making mechanisms. Knowledge management is the process that enables knowledge to be acquired, stored, disseminated, used and developed, allowing members of an organization to apply knowledge. According to other experts, knowledge management is a process of knowledge exchange within an organization but also a set of processes of formal and informal networks of knowledge exchange between organizations or even sectors, which facilitate intersectoral cooperation, improve organizational performance and solve complex problems.

**The definition of a knowledge broker**

The term knowledge broker is used synonymously with several terms in the international literature, such as innovation broker, knowledge intermediary, technology broker, and change agent. Some authors use the term knowledge broker to refer to organizations rather than individuals, and the following terms are also used synonymously in the literature: intermediary firms, boundary organizations, or bridging institutions. Knowledge brokers, whether individuals or organizations, facilitate the interpretation, sharing and application of knowledge by acting as intermediaries of knowledge, information and experience between two or more actors (such as individuals, organizations, communities or networks). Some have argued that knowledge brokers play an essential role in the knowledge-producing research community and the knowledge users (stakeholders), stimulating the emergence of new information, collaborative knowledge exchange, and evidence-based approaches. Knowledge Brokers' activities include building and operating partnerships and networks; identifying and connecting key actors; identifying, evaluating and analyzing relevant information; supporting communication and information sharing; developing analytical and interpretative skills; and producing knowledge products.

**Current activities and experiences in knowledge management and knowledge brokering in Health Promotion Offices**

- Based on the definition of knowledge management, are there any activities you are currently implementing in the Health Promotion Offices? Can you give some examples?

- Based on the definition of knowledge brokering, are there any activities you are currently implementing in Health Promotion Offices? Can you give examples?

**The possibilities of Health Promotion Offices for knowledge brokering and supporting evidence-based decision-making**

- In your opinion, would it be useful if the activities of the Health Promotion Offices were to include more emphasis on knowledge management or knowledge brokering?

- If so, for which topics or areas would you consider it useful to have a more pronounced knowledge management activities or knowledge brokering function?

- Could Health Promotion Offices as knowledge brokers promote the spread of evidence-based decision-making at the municipal or district level in the planning, implementation and evaluation of health-related interventions?

**Factors hindering effective knowledge management activities and knowledge broker function**

- What factors prevent Health Promotion Offices from being able to carry out knowledge management activities effectively?

- What factors prevent Health Promotion Offices from being able to perform knowledge brokerage activities effectively?

**Factors supporting effective knowledge management activities and the knowledge broker function**

- What factors support the ability of Health Promotion Offices to carry out knowledge management activities effectively?

- Which factors support the ability of Health Promotion Offices to perform knowledge brokerage activities effectively?

**Concluding the interview**

- Are there any thoughts that have not been expressed but that you think are important to share on this topic?
