# Supplementary material for: Knowledge management and knowledge brokering in the Health Promotion Offices in Hungary: a qualitative study
Source: Front Public Health. 2025 Jun 9;13:1588370. doi: 10.3389/fpubh.2025.1588370 (PMC12183307; doi:10.3389/fpubh.2025.1588370)
Supplement: Supplementary file 3 [file Table_3.docx]

Appendix 3. Characteristics of interview partners.

| **ID** | **Gender** | **Region** | **Duration of interview (hh:mm)** |
| --- | --- | --- | --- |
| 1 | male | Central Transdanubia | 0:45 |
| 2 | female | Capital | 0:58 |
| 3 | female | Southern Great Plain | 1:05 |
| 4 | female | Western Transdanubia | 0:59 |
| 5 | male | Central Hungary | 1:10 |
| 6 | female | Southern Great Plain | 1:04 |
| 7 | male | Central Transdanubia | 0:47 |
| 8 | female | Western Transdanubia | 0:53 |
| 9 | male | Northern Great Plain | 0:46 |
| 10 | male | Southern Great Plain | 0:46 |
| 11 | male | Southern Transdanubia | 0:45 |
| 12 | female | Northern Great Plain | 0:57 |
| 13 | male | Capital | 1:19 |
| 14 | female | Northern Great Plain | 0:48 |
| 15 | male | Central Hungary | 1:12 |
| 16 | male | Capital | 1:03 |
| 17 | female | Northern Hungary | 0:54 |
| 18 | female | Capital | 0:48 |
| 19 | male | Western Transdanubia | 0:53 |
| 20 | female | Northern Hungary | 0:44 |
| 21 | male | Central Hungary | 0:40 |
| 22 | female | Southern Transdanubia | 0:59 |
